# Eco-Friendly Synthesis of Some Thiosemicarbazones and Their Applications as Intermediates for 5-Arylazothiazole Disperse Dyes

**DOI:** 10.3390/molecules201219820

**Published:** 2015-12-09

**Authors:** Hatem E. Gaffer, Mohamed E. Khalifa

**Affiliations:** 1 Textile Research Division, National Research Center, Dokki 12622, Egypt; hatem197@yahoo.com; 2 Department of Chemistry, Faculty of Science, Taif University, P. O. Box 888, Taif 21974, Saudi Arabia; 3 Department of Chemical Engineering, Higher Institute for Engineering and Technology, Central Zone, 4th District, New Damietta, Damietta, Egypt

**Keywords:** solid-solid reactions, thiosemicarbazones, phenacyl bromide, 5-arylazothiazoles, disperse dyes, fastness properties.

## Abstract

The solid-solid reactions of thiosemicarbazide with 4-formylantipyrine, 2-acetylpyrrole and camphor were performed to afford the thiosemicarbazones **1**–**3** which underwent hetero-cyclization with phenacyl bromide to furnish the corresponding thiazole derivatives **4**–**6**. The yields of the reactions are quantitative in all cases and the products do not require further purification. A series of 5-arylazo-2-(substituted ylidene-hydrazinyl)-thiazole dyes **7**–**9** was then prepared by diazo coupling of thiazole derivatives **4**–**6** with several diazonium chlorides. The synthesized dyes were applied as disperse dyes for dyeing polyester fabric. The dyed fabrics exhibit good washing, perspiration, sublimation and light fastness properties, with little variation in their moderate to good rubbing fastness.

## 1. Introduction

Stoichiometric solid-solid reactions are most versatile for the waste-free production of a single product, while melt reactions more frequently have the risk of side reactions, even if these are considerably better than solution reactions. Solvent-free reactions have been reviewed without emphasis of waste-free processes [1]. More than 1000 waste-free quantitative syntheses in organic solid-state chemistry are already known and have been reported in a review article that covers more than 25 reaction types [2]. Thiosemicarbazones are a class of interesting compounds presenting a wide range of pharmacological applications as antitumor, antimicrobial and antiviral agents [3]. α-(*N*)-Heterocyclic thiosemicarbazones have been extensively investigated by many authors [4,5,6,7,8,9].

## 2. Results and Discussion

### 2.1. Chemistry

**Scheme 1:**
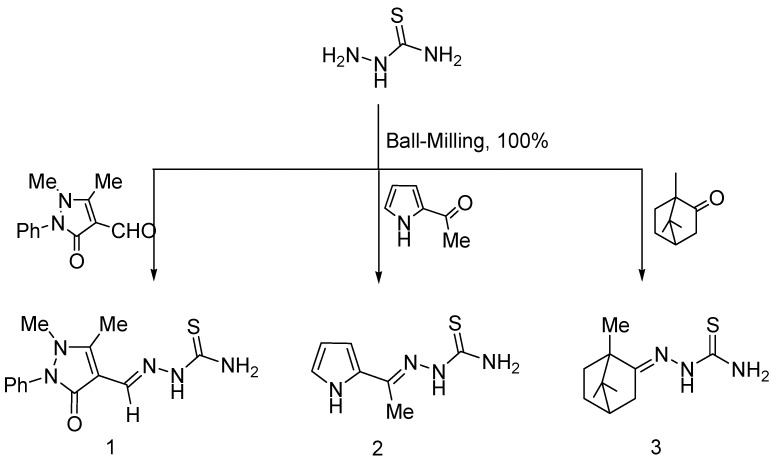
Synthesis of thiosemicarbazones **1**, **2** and **3** using the ball-milling technique at room temperature.

Thiosemicarbazones **1**–**3** were prepared quantitatively by room temperature ball-milling thiosemicarbazide with 4-formylantipyrine, 2-acetylpyrrole and camphor, respectively (Scheme 1). The water of reaction can be removed by heating to 80 °C in a vacuum (Scheme 1). The solid-state condensation is quantitative and more effective than the reported solution reactions (**1**, 88% [10]; **2**, 56% [11]; **3**, 85% [12]). We have been able to increase the yield in various instances from moderate in solution to quantitative by applying solid–solid techniques without any solvent [13,14,15]. Thus, thio- semicarbazones are now most readily available without producing wastes from versatile condensations of thiosemicarbazides and aldehydes or ketones. We present here the synthetic use of these environmentally benign building blocks in various heterocyclization reactions.

**Scheme 2:**
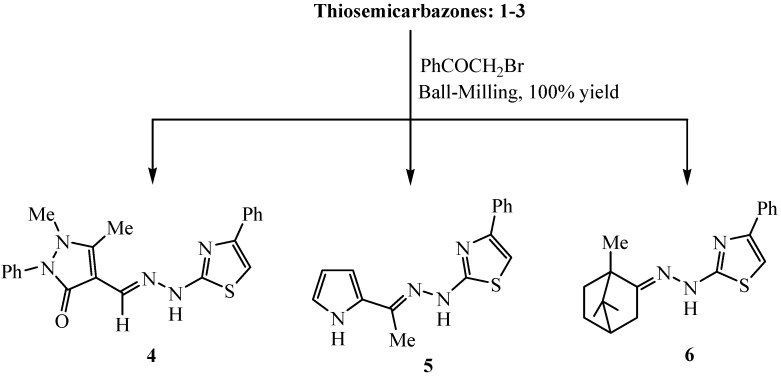
Synthesis of 2-(substituted ylidene-hydrazinyl)-4-phenylthiazoles **4**, **5** and **6**.

**Scheme 3:**
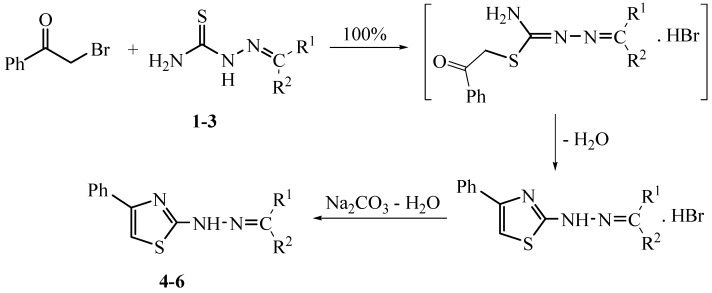
The suggested solid-state mechanism for the reaction of phenacyl bromide and thiosemicarbazones **1**–**6**.

Thiosemicarbazides are convenient precursors which have been extensively utilized in heterocyclic synthesis [16]. Their reactions with compounds containing C=O and C=N groups is an elegant method for the preparation of biologically active compounds *viz*. triazoles and thiazoles [17]. The waste-free solid-state reaction technique could also be applied to prepare the 2-(substituted ylidene-hydrazinyl)-4-phenylthiazoles **4**, **5** and **6**. Stoichiometric ball milling reactions of phenacyl bromide with 4-formylantipyrine thiosemicarbazone (**1**), 2-acetylpyrrole thiosemicarbazone (**2**) and camphor thiosemicarbazone (**3**) afforded the corresponding iminium hydrobromide salts in 100% yield without the aid of basic catalysts or solvents. The water of the reaction can be removed by evaporation at 80 °C under vacuum without hydrolysis losses. Washing with aqueous Na_2_CO_3_ can easily liberate the 2-(substituted ylidene-hydrazinyl)-4-phenylthiazole free bases **4**, **5** and **6** (Scheme 2). The suggested mechanism for the formation of compounds **4**, **5** and **6** is outlined in Scheme 3 and in agreement with previously reported results [18]. It is thought that the reaction starts through nucleophilic attack of the thiolate group to form the non-isolable S-alkylated intermediate, which via nucleophilic addition and intramolecular cyclocondensation accompanied by water elimination, gave the corresponding thiazoles **4**, **5** and **6**. The chemical structures of **4**–**6** were elucidated on the basis of spectral techniques. The IR spectrum of **4** (for example) showed the characteristic absorption bands for NH, C=N and C=O stretching at 3121, 1614 and 1659 cm^−1^. The ^1^H-NMR spectrum of **4** displayed two singlet signals of six protons for two methyl groups at δ 2.55 and 3.20 ppm, three singlet protons at δ 7.15, 8.35, and 9.60 ppm due to the thiazole H-5, azomethine proton (CH=N), and NH proton, in addition to a multiplet signal centered around the region 7.25–7.75 assigned to the ten aromatic protons of two phenyl rings. The mass spectrum of **4** showed a molecular ion peak at *m*/*z* = 389 (intensity 100%) corresponding to the correct molecular weight for the molecular formula C_21_H_19_N_5_OS.

**Scheme 4:**
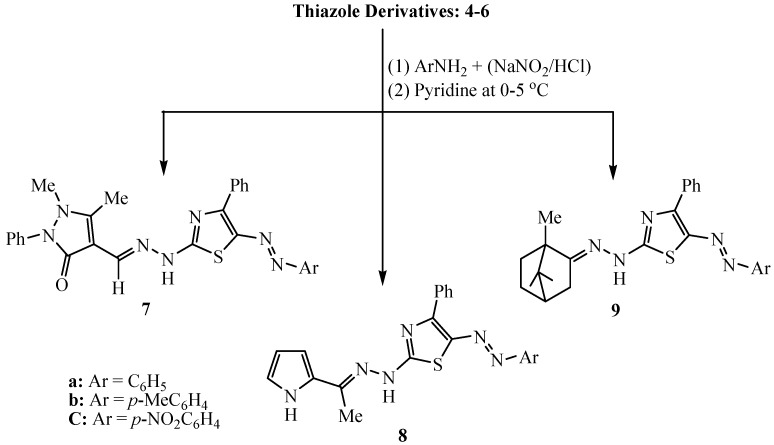
Synthesis of 5-arylazo-4-phenylthiazole derivatives **7**, **8** and **9**.

Diazo coupling of the synthesized 2-(substituted ylidene-hydrazinyl)-4-phenylthiazoles **4**, **5** and **6** with several aromatic diazonium chlorides proceeded in pyridine at 0–5 °C to afford the corresponding 5-arylazo-4-phenylthiazole derivatives **7**, **8** and **9** respectively (Scheme 4).

The structures of the highly functionalized thiazolyl dyes **7**–**9** were assigned on the basis of their elemental analyses and spectral data. For example, the IR spectrum of **7a** exhibited the presence of NH, C=O and C=N stretching bands at 3361, 1678 and 1632 cm^−1^, respectively. The ^1^H-NMR spectrum of **7a** revealed two singlet signals at δ 2.45 and 3.35 ppm corresponding to six protons of two methyl groups (2CH_3_), a multiplet in the region δ 6.95–7.80 ppm due to aromatic protons, a singlet signal at δ 8.30 ppm in due to methine proton (CH=N), in addition to a singlet signal at δ 11.80 ppm due to NH proton. The mass spectrum of **7a** (Ar = C_6_H_5_) showed a molecular ion peak at *m*/*z* = 493 (intensity 81.7%) corresponding to the correct molecular weight for the molecular formula C_27_H_23_N_7_OS.

### 2.2. Application

#### 2.2.1. Dyeing and Fastness Properties

For dyeing polyester fabrics, in practical terms, only disperse dyes are suitable. Through their hydrophobic properties, these dyes are capable of penetrating into the similarly hydrophobic polyester fiber. The functionalized 5-arylazo-4-phenyl-2-substituted-thiazole disperse dyes **7**–**9** were applied to polyester fabrics (o.w.f. 2%) by the high-temperature pressure technique (130 °C) and a range of color shades ranging from red to violet has been obtained. The dyes obtained gave excellent leveling, uniformity of coloration and exhaustion of dye liquor. The dyeing of polyester fabrics was evaluated in terms of their fastness properties (e.g., fastness to washing, perspiration, rubbing, sublimation, and light) using a standard method [19]. The results are given in Table 1 and they reveal that these dyes share good fastness properties.

**Table 1:** Fastness properties of the synthesized dyes on polyester fabrics.

Dye	Washing	Perspiration		Rubbing		Sublimation		Light (60 h)
		Acid	Alkali	Dry	Wet	Staining at 180 °C	Staining at 210 °C	
**7a**	4–5	4	4–5	4–5	4–5	3–4	3	5–6
**7b**	4–5	4–5	4–5	3	3	4	4	3–4
**7c**	4–5	4	4–5	3–4	4–5	4	4	6
**8a**	4–5	4	4	3–4	4	3–4	3	6
**8b**	4–5	4–5	4	4	4	3–4	4	4
**8c**	4–5	4–5	4–5	3	3	4	3–4	5–6
**9a**	4–5	4	4	3	3	4	4	5–6
**9b**	4–5	4–5	4	4	4–5	4	3–4	4
**9c**	4–5	4–5	4–5	4	4–5	4	4	6

The light fastness of the synthesized dyes on polyester is significantly affected by the nature of substituent in the diazo component, where the fading of azo dyes is mainly a consequence of decomposition of the -N=N- moiety by oxidation, reduction and/or photolysis [20]. The rates of these processes should be sensitive to the chemical structure of the dye, the type of substrate and treatment conditions. Since the dyed substrate employed in this study is a polyester fabric (*i.e.*, non-proteinic), the fading process likely occurs by oxidation [21]. The ease of oxidation of azo linkages should be a function of electron density. Therefore, electron-donating substituent on this moiety should increase the fading rate. This proposal is in agreement with the observed results (Table 1) which demonstrate that the presence of a methyl group in the synthesized dyes causes a decrease of light fastness to 3–4.

#### 2.2.2. Color Assessment

The color on polyester fibers is expressed in terms of CIELAB values (Table 2) and the following CIELAB coordinates are measured: lightness (L*), chroma (C*), hue angle (h) from 0° to 360°, (a*) value represents the degree of redness (positive) and greenness (negative) and (b*) represents the degree of yellowness (positive) and blueness (negative). K/S values given by the reflectance spectrometer are calculated at λ_max_ and are directly correlated with the dye concentration on the dye substrate according to the Kubelka–Munk equation: K/S = (1 − R)^2^/2R, where K = absorbance coefficient, S = scattering coefficient, R = reflectance ratio. The difference in color strength depends on the substitutes present and/or the position of the substitutes on the structure of the synthesized dyes. The presence of bathochromes “electron-donating substitutes” such as methyl group (*i.e.*, **7b**, **8b** and **9b**) deepen the color due to their bathochromic effect, whereas presence of hypochromes “electron-attracting substitutes” such as nitro group (*i.e.*, **7c**, **8c** and **9c**) make the opposite effect on color strength due to their hypsochromic shift.

As shown in Table 2, application of the synthesized compounds **7(a**–**c)**–**9(a**–**c)** as disperse dyes, showed the good affinity of such dyes to polyester fabrics, as indicated from the satisfactory color yields and the acceptable K/S values.

**Table 2:** Optical measurements of the synthesized dyes on polyester fabrics.

Dye	Exhaustion (%)	Molar Absorbitivity ɛ	Absorption λ_max_/nm	K/S	L*	a*	b*	C*	H
**7a**	61	1.42 × 10^5^	451	10.31	81.07	3.34	20.41	51.03	67.49
**7b**	64	1.18 × 10^5^	417	13.87	40.99	3.32	41.81	44.88	66.24
**7c**	61	1.89 × 10^4^	447	10.23	74.23	−1.23	37.20	55.34	80.62
**8a**	62	1.17 × 10^5^	453	11.24	89.36	5.35	3.14	53.62	76.96
**8b**	62	1.22 × 10^5^	444	11.46	72.91	5.22	47.57	48.46	82.52
**8c**	65	2.11 × 10^5^	452	13.72	73.22	0.37	23.11	47.56	84.88
**9a**	62	1.08 × 10^5^	464	11.07	65.51	−1.44	30.77	44.39	80.63
**9b**	63	1.17 × 10^5^	451	11.57	80.93	−1.05	14.02	51.74	74.65
**9c**	65	1.41 × 10^5^	443	11.68	80.34	−0.56	16.23	55.23	66.09

**Figure 1:**
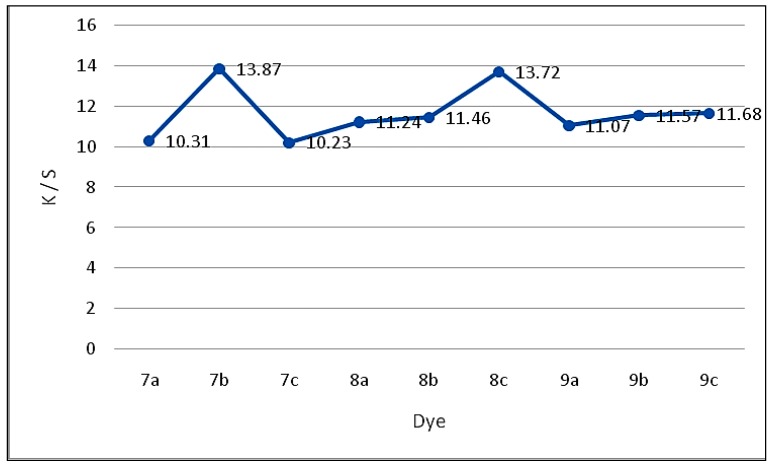
Comparison of K/S values for the synthesized disperse dyes **7**–**9** series.

Figure 1 illustrates the relationship between the dyeing bath concentrations and the strengths of the synthesized **7(a**–**c)**–**9(a**–**c)** disperse dyes (K/S), under high temperature (HT) dyeing conditions, where no direct correlation between the relative molecular mass of these dyes to the build-up was apparent. This probably reflects the importance of other parameters such as dye solubility, which in turn is dictated by the type of structural features within the dye. These results are in line with the previously reported by Müller [22] on the relation between substituents and dye structure.

In general, the color hues of the thiazolyl dyes **7(a**–**c)**–**9(a**–**c)** on polyester fabric are shifted to the greenish direction, as indicated by the negative values of a* (red-green axis). The positive values of b* (yellow-blue axis) indicate that the color hues of the thiazolyl—azo dyes **7a**–**c**, **8a**–**c** and **9a**–**c** on polyester fabric are shifted to the yellowish direction.

## 3. Experimental Section

### 3.1. Materials and Methods

#### 3.1.1. Chemicals and Reagents

All the chemicals and solvents used in this study were obtained from Merck (Darmstadt, Germany) and Sigma-Aldrich chemical company (St. Louis, MO, USA).

#### 3.1.2. Instrumentation

Melting points were determined with a Gallenkamp melting point apparatus (capillary method, Gallenkamp Co., London, UK) and are uncorrected. Elemental analyses were carried out at the Microanalytical Unit of National Research Center and Faculty of Science, Mansoura University; the results were in satisfactory agreement with the calculated values. IR spectra (KBr) were recorded with a Matteson 5000 FTIR spectrometer (Shimadzu Co., Kyoto, Japan, not all frequencies are reported). The ^1^H-NMR spectra were acquired using a WP 300 spectrometer (Bruker Co., Billerica, MA, USA) at 300 MHz (1H) in broad band mode. Mass spectra were obtained at a Finnigan MAT 212 instrument (Finnigan MAT Co., Bremen, Germany) by electron impact at 70 eV. The ball-mill was a Retsch MM 2000 swing mill (Retsch, Haan, Germany) with a 10-mL stainless steel, double-walled beaker with fittings for circulating coolants. Two stainless steel balls of 12 mm diameter were used. Ball-milling was performed at 20–25 Hz frequency, usually at room temperature (without circulating liquid the temperature did not rise above 30 °C). The colorimetric measurements for the dyed polyester fabrics were carried out using a reflectance spectrophotometer (GretagMacbeth CE 7000a, GretagMacbeth Co., Windsor, UK). Fastness to washing was carried out using the automatic Rotadyer launder (sponsored by the British Standard Institute—Society of Dyers and Colourists), fastness to perspiration was assessed according to the test sponsored by the (BSS), fastness to rubbing was carried out according to the standard method of testing (BSS) using a Crockmeter FD-17 apparatus (Electric Hungarian Co., Budapest, Hungary), fastness to sublimation was carried out using scorch tester M247 A (Atlas Electric Devices Co., Chicago, IL, USA) and fastness to light was carried out using the “Weather-o-meter” (Atlas Electric Devices Co., Chicago, IL, USA) according to the AATCC standard test method.

### 3.2. Synthesis

#### 3.2.1. Solid-State Synthesis of Thiosemicarbazones **1**–**3**

A mixture of thiosemicarbazide (0.182 g, 2.00 mmol) and 2.00 mmol of 4-formylantipyrine, 2-acetylpyrrole or camphor was ball-milled at room temperature for 1 h. The resulting solid powders were dried at 80 °C under vacuum to give a 100% yield of the corresponding thiosemicarbazones **1**, **2** and **3** that did not require any further purification.

*4-Formylantipyrine thiosemicarbazone* (**1**). White solid; yield 100%; m.p. 219–220 °C (Lit. yield 88%; m.p. 217–219 °C [10]); IR: ῡ = 3378, 3236 (NH and NH_2_), 1682 (C=O) cm^−1^; ^1^H-NMR (CDCl_3_): δ = 2.45 (s, 3H, CH_3_), 3.20 (s, 3H, CH_3_N), 7.00–7.45 (m, 5H, Ar-H), 8.00 (s, 2H, NH_2_), 8.40 (s, 1H, CH=N), 9.55 (s, 1H, NH). Anal. Calcd. for C_13_H_15_N_5_OS (289.36): C, 53.96; H, 5.23; N, 24.20%. Found: C, 53.83; H, 5.28; N, 24.32%.

*2-Acetylpyrrole thiosemicarbazone* (**2**). White solid; yield 100%; m.p. 192–193 °C (Lit. yield 56%; m.p. 193 °C [11]); IR: ῡ= 1642 (C=N), 3376, 3268, 3188 (NH and NH_2_) cm^−1^; ^1^H-NMR (CDCl_3_): δ = 2.20 (s, 3H, CH_3_), 6.05 (q, 1H, pyrrole H-4), 6.50 (d, 1H, pyrrole H-3), 6.90 (d, 1H, pyrrole H-5), 8.25 (s, 2H, NH_2_), 9.90 (s, 1H, NH), 11.45 (s, 1H, NH). Anal. Calcd. for C_7_H_10_N_4_S (182.25): C, 46.13; H, 5.53; N, 30.74%. Found: C, 46.34; H, 5.48; N, 30.62%.

*Camphor thiosemicarbazone* (**3**). Yellow solid; yield 100%; m.p. 148–150 °C (Lit. yield 85%; m.p. 147–150 °C [12]); IR: ῡ= 1590 (C=N), 3440, 3241, 3162 (NH and NH_2_) cm^−1^; ^1^H-NMR (CDCl_3_): δ = 0.75 (s, 3H, CH_3_), 0.95 (s, 6H, 2CH_3_), 1.20–1.30 (m, 2H, H-5 and H-6 endo), 1.70–1.80 (m, 2H, H-5 and H-6 exo), 1.90–2.00 (m, 2H, H-3 endo and H-4), 2.35–2.40 (m, 1H, H-3 exo), 8.00 (s, 2H, NH_2_), 9.70 (s, 1H, NH). Anal. Calcd. for C_11_H_19_N_3_S (225.35): C, 58.63; H, 8.50; N, 18.65%. Found: C, 58.77; H, 8.57; N, 18.56%.

#### 3.2.2. Solid-State Synthesis of Thiazole Derivatives **4**–**6**

A mixture of phenacyl bromide (0.398 g, 2.00 mmol) and thiosemicarbazone derivatives **1**, **2**, or **3** (2.00 mmol) was ball-milled at room temperature for 1 h. After drying at 0.01 bar at 80 °C quantitative yields of the hydrobromide salts were obtained. The free bases **4**–**6** were obtained by washing the fine powder of the corresponding hydrobromide salts with 5% Na_2_CO_3_ solution, followed by water and drying at 80 °C under vacuum.

*2-(Antipyrin-4-ylmethylidenehydrazinyl)-4-phenylthiazole* (**4**). Yellow solid; yield 98%; m.p. 235–237 °C (Lit. yield 68%; m.p. 234–235 °C [23]); IR: ῡ= 3121(NH), 1614 (C=N), 1659 (C=O) cm^−1^; ^1^H-NMR (CDCl_3_/DMSO): δ = 2.55 (s, 3H, CH3), 3.20 (s, 3H, CH3), 7.15 (s, 1H, thiazole H-5), 7.25–7.75 (m, 10H, Ar-H and), 8.35 (s, 1H, CH=N), 9.60 (s, 1H, NH). MS (*m/z*, relative intensity): 389 (M^+^, 100%). Anal. Calcd. for C_21_H_19_N_5_OS (389.47): C, 64.76; H, 4.92; N, 17.98%. Found: C, 64.55; H, 4.84; N, 17.86%.

*2-[2-(1-(1H-Pyrrol-2-yl)ethylidene)hydrazinyl]-4-phenylthiazole* (**5**). Yellow solid; yield 95%; m.p. 212–213 °C (EtOH); IR: ῡ= 3147 (NH), 1616 (C=N) cm^−1^; ^1^H-NMR (CDCl_3_): δ = 2.15 (s, 3H, CH_3_), 6.11–6.90 (m, 3H, pyrrole), 7.25 (s, 1H, thiazole H-5), 7.42–8.13 (m, 6H, Ar-H + NH), 9.65 (s, 1H, NH). MS (*m/z*, relative intensity): 282 (M^+^, 88%). Anal. Calcd. for C_15_H_14_N_4_S (282.36): C, 63.80; H, 5.00; N, 19.84%. Found: C, 63.91; H, 5.08; N, 19.72%.

*4-Phenyl-2-[2-(1,7,7-trimethylbicyclo[2.2.1]heptan-2-ylidene)hydrazinyl]thiazole* (**6**). Yellow solid; yield 97%; m.p. 254–255 °C (EtOH); IR: ῡ= 3168 (NH), 1609 (C=N) cm^−1^; ^1^H-NMR (CDCl_3_): δ = 0.90–1.05 (s, 9H, 3CH_3_), 1.30 (m, 2H, CH_2_), 1.70 (m, 2H, CH_2_), 1.90 (m, 2H, CH_2_), 2.30 (m, 1H, CH), 7.10–7.65 (m, 6H, Ar-H + thiazole H-5), 9.20 (s, 1H, NH). MS (*m/z*, relative intensity): 326 (M^+^ + 1, 100%). Anal. Cacld. for C_19_H_23_N_3_S (325.47): C, 70.11; H, 7.12; N, 12.91%. Found: C, 70.27; H, 7.03; N, 13.03%.

#### 3.2.3. Synthesis of 5-Arylazo-2-(arylidenehydrazino)-4-phenyl-thiazole Dyes **7**–**9**

A cold solution of sodium nitrite (2 mmol) in water (5 mL) was added gradually to a cold suspension of desired aromatic amine (2 mmol) in concentrated HCl (0.6 mL). The diazonium salt thus obtained was added by continuous stirring to a cold solution of compound **4**, **5** and/or **6** (2 mmol) in 10 mL of pyridine. The reaction mixture was stirred at 0–5 °C for 2 h and diluted with water. The solid was then filtered, dried, and recrystallized from ethanol or an ethanol-DMF mixture to afford the corresponding 5-arylazothiazole derivatives **7**, **8** and/or **9**, respectively.

*2-(Antipyrin-4-ylmethylidenehydrazinyl)-4-phenyl-5-phenylazo-thiazole* (**7a**). Red solid; yield 87%; m.p. 185–86 °C (EtOH); IR: ῡ= 3361 (NH), 1678 (C=O) and 1632 (C=N) cm^−1^; ^1^H-NMR (DMSO): δ = 2.45 (s, 3H, CH_3_), 3.35 (s, 3H, CH_3_N), 6.95–7.80 (m, 15H, Ar-H), 8.30 (s, 1H, CH=N), 11.80 (s, 1H, NH). MS (*m*/*z*, relative intensity): 493 (M^+^, 81%). Anal. Calcd. for C_27_H_23_N_7_OS (393.58): C, 65.70; H, 4.70; N, 19.86%. Found: C, 65.62; H, 4.66; N, 19.80%.

*2-(Antipyrin-4-ylmethylidenehydrazinyl)-4-phenyl-5-(p-tolylazo)-thiazole* (**7b**). Red solid; yield 76%; m.p. 196–197 °C (EtOH); IR: ῡ= 3346 (NH), 1677 (C=O) and 1628 (C=N) cm^−1^; ^1^H-NMR (DMSO): δ = 2.30 (s, 3H, CH_3_), 2.50 (s, 3H, CH_3_), 3.35 (s, 3H, CH_3_N), 7.05–7.80 (m, 14H, Ar-H), 8.25 (s, 1H, CH=N), 11.20 (s, 1H, NH). MS (*m/z*, relative intensity): 508 (M^+^ + 1, 76%). Anal. Calcd. for C_28_H_25_N_7_OS (507.61): C, 66.25; H, 4.96; N, 19.32%. Found: C, 66.14; H, 4.90; N, 19.41%.

*2-(Antipyrin-4-ylmethylidenehydrazinyl)-4-phenyl-5-(p-nitrophenlylazo)-thiazole* (**7c**). Reddish brown solid; yield 82%; m.p. 257–258 °C (EtOH-DMF); IR: ῡ= 3324 (NH), 1672 (C=O) and 1635 (C=N) cm^−1^; ^1^H-NMR (DMSO): δ = 2.45 (s, 3H, CH_3_), 3.35 (s, 3H, CH_3_N), 7.10–8.10 (m, 14H, Ar-H), 8.25 (s, 1H, CH=N), 11.65 (s, 1H, NH). MS (*m*/*z*, relative intensity): 539 (M^+^ + 1, 58%). Anal. Calcd for C_27_H_22_N_8_O_3_S (538.58): C, 60.21; H, 4.12; N, 20.81%. Found: C, 60.16; H, 4.03; N, 20.74%.

*2-[2-(1-(1H-Pyrrol-2-yl)ethylidene)hydrazinyl]-4-phenyl-5-phenylazothiazole* (**8a**). Reddish brown solid; yield 65%; m.p. 172–173 °C (EtOH); IR: ῡ= 3368, 3272 (NH), 1641 (C=N), cm^−1^; ^1^H-NMR (DMSO): δ = 2.20 (s, 3H, CH_3_), 6.30–6.90 (m, 3H, pyrrole), 7.35–7.90 (m, 10H, Ar-H), 9.40 (s, 1H, NH), 11.55 (s, 1H, NH). MS (*m/z*, relative intensity): 386 (M^+^, 92%). Anal. Calcd. for C_21_H_18_N_6_S (386.47): C, 65.26; H, 4.69; N, 21.75%. Found: C, 65.42; H, 4.61; N, 21.79%.

*2-[2-(1-(1H-Pyrrol-2-yl)ethylidene)hydrazinyl]-4-phenyl-5-(4-methylphenyl)azothiazole* (**8b**). Reddish brown solid; yield 78%; m.p. 247–248 °C (EtOH-DMF); IR: ῡ= 3344, 3234 (NH ), 1637 (C=N), cm^−1^; ^1^H-NMR (DMSO): δ = 2.25 (s, 3H, CH_3_), 2.40 (s, 3H, CH_3_), 6.30–6.80 (m, 3H, pyrrole), 7.20–7.85 (m, 9H, Ar-H), 9.55 (s, 1H, NH), 11.40 (s, 1H, NH). MS (*m/z*, relative intensity): 400 (M^+^, 74%). Anal. Calcd for C_22_H_20_N_6_S (400.50): C, 65.98; H, 5.03; N, 20.98%. Found: C, 65.82; H, 5.11; N, 20.87%.

*2-[2-(1-(1H-Pyrrol-2-yl)ethylidene)hydrazinyl]-4-phenyl-5-(4-nitrophenyl)azothiazole* (**8c**). Violet solid; yield 80%; m.p. 237–238 °C (EtOH); IR: ῡ= 3334, 3254 (NH), 1651 (C=N) cm^−1^; ^1^H-NMR (DMSO): δ = 2.25 (s, 3H, CH_3_), 6.30–6.80 (m, 3H, pyrrole), 7.30–8.10 (m, 9H, Ar-H), 9.15 (s, 1H, NH), 11.35 (s, 1H, NH). MS (*m/z*, relative intensity): 431 (M^+^, 68%). Anal. Calcd for C_21_H_17_N_7_S (431.47): C, 58.46; H, 3.97; N, 22.72%. Found: C, 58.38; H, 3.93; N, 22.67%.

*4-Phenyl-5-phenylazo-2-[2-(1,7,7-trimethylbicyclo[2.2.1]heptan-2-ylidene)-hydrazinyl]thiazole* (**9a**). Red solid; yield 87%; m.p. 201–202 °C (EtOH-DMF); IR: ῡ= 3272 (NH), 1631 (C=N) cm^−1^; ^1^H-NMR (DMSO): δ = 0.90–1.00 (s, 9H, 3CH_3_), 1.25 (m, 2H, CH_2_), 1.70 (m, 2H, CH_2_), 1.90 (m, 2H, CH_2_), 2.20 (m, 1H, CH), 7.10–7.75 (m, 10H, Ar-H), 11.15 (s, 1H, NH). Anal. Calcd. for C_25_H_27_N_5_S (429.58): C, 69.90; H, 6.34; N, 16.30%. Found: C, 69.81; H, 6.28; N, 16.23%.

*4-Phenyl-2-[2-(1,7,7-trimethylbicyclo[2.2.1]heptan-2-ylidene)hydrazinyl]-5-(p-tolylazo)thiazole* (**9b**). Red solid; yield 81%; m.p. 214–215 °C (EtOH-DMF); IR: ῡ= 3268 (NH), 1627 (C=N) cm^−1^; ^1^H-NMR (DMSO): δ = 0.90–1.05 (s, 9H, 3CH_3_), 1.20 (m, 2H, CH_2_), 1.70 (m, 2H, CH_2_), 1.90 (m, 2H, CH_2_), 2.20 (m, 1H, CH), 2.40 (s, 3H, CH_3_), 7.05–7.75 (m, 9H, Ar-H), 10.85 (s, 1H, NH). Anal. Calcd. for C_26_H_29_N_5_S (443.61): C, 70.40; H, 6.59; N, 15.79%. Found: C, 70.30; H, 6.47; N, 15.64%.

*4-Phenyl-2-[2-(1,7,7-trimethylbicyclo[2.2.1]heptan-2-ylidene)hydrazinyl]-5-(p-nitrophenylazo)thiazole* (**9c**). Violet solid; yield 88%; m.p. 274–275 °C (EtOH-DMF); IR: ῡ= 3272 (NH), 1647 (C=N) cm^−1^; ^1^H-NMR (DMSO): 0.90–1.05 (s, 9H, 3CH_3_), 1.30 (m, 2H, CH_2_), 1.70 (m, 2H, CH_2_), 1.90 (m, 2H, CH_2_), 2.25 (m, 1H, CH), 7.15–8.10 (m, 9H, Ar-H), 11.25 (s, 1H, NH). Anal. Calcd. for C_25_H_26_N_6_O_2_S (474.58): C, 63.27; H, 5.52; N, 17.71%. Found: C, 63.18; H, 5.43; N, 17.67%.

### 3.3. Dyebath Preparation and Dyeing Procedure

The synthesized disperse dyes under investigation **7**–**9** series, were applied to polyester fabrics (0.04 g dye/2 g fabric; 2% shade) by a convenient method for dyeing polyester fabrics in the laboratory at 130 °C and high pressure was applied. Dyes were dispersed by dissolving the appropriate amount of dye in acetone (1 mL) and then added dropwise with stirring to the dyebath (liquor ration 20:1) containing an anionic dispersing agent such as 1% Setamol WS from BASF. After adjusting THE pH of the dyebath at 5.5 using aqueous acetic acid the wetted-out polyester fibers were added. Dyeing was performed by raising the dyebath temperature from 20 to 130 °C at a rate of 3 °C/min and then maintained for 60 min., followed by rapid cooling (9.9 °C/min.) to 50 °C. the dyed fabrics were rinsed with cold water and reduction cleared (1 g/L sodium hydroxide, 1 g/L sodium hydrosulfite, 10 min, 80 °C). The samples were rinsed with hot and cold water and finally air-dried.

### 3.4. Dyeing Characteristics on Polyester Fabrics

#### 3.4.1. Color Fastness Tests

The color fastness properties of the dyed fibres to washing, perspiration, rubbing, sublimation and light were evaluated using standard methods [19]. The staining of adjacent cotton and nylon fabrics was assessed using the grey scale: 1-poor, 2-fair, 3-moderate, 4-good and 5-excellent, other than light fastness which scaled from 1–8 on the grey scale.

#### 3.4.2. Color Properties of the Dyes on Polyester

A reflectance spectrophotometer (GretagMacbeth CE 7000a; D65 illumination, 10° observer) was used for the colorimetric measurements on the dyed samples. K/S values given by the reflectance spectrometer are calculated at λ_max_ and are directly correlated with the dye concentration on the dye substrate according to the Kubelka–Munk equation K/S = (1 − R)^2^/2R, where K = absorbance coefficient, S = scattering coefficient, R = reflectance ratio.

## 4. Conclusions

A set of novel 5-arylazo-2-(substituted ylidene-hydrazinyl)thiazole disperse dyes was synthesized from novel thiazole derivatives prepared in quantitative yields by the reaction of versatile thiosemicarbazones and phenacyl bromide using a ball-milling solid-solid reaction technique. The newly synthesized dyes were applied as disperse dyes for dyeing polyester fabric, where they exhibited very good dyability and fastness properties.

## References

[1] Tanaka K. (2009). Solvent-Free Organic Synthesis.

[2] Kaupp G., Toda F. (2005). Organic solid-state reactions with 100% yield. Organic Solid State Reactions.

[3] Beraldo H., Gambinob D. (2004). The wide pharmacological versatility of semicarbazones, thiosemicarbazones and their metal complexes. Mini Rev. Med. Chem..

[4] Altun A., Kumru M., Dimoglo A. (2001). Study of electronic and structural features of thiosemicarbazone and thiosemicarbazide derivatives demonstrating anti-HSV-1 activity. J. Mol. Struct..

[5] Cory J.G., Cory A.H., Rappa G., Lorico A., Mao-Chin L., Tai-Shun L., Sartorelli A.C. (1994). Inhibitors of ribonucleotide reductase: Comparative effects of amino- and hydroxy-substituted pyridine-2-carboxaldehydethiosemicarbazones. Biochem. Pharmacol..

[6] Liu M.C., Lin T-S., Cory J.G., Cory A.H., Sartorelli A.C. (1996). Synthesis and biological activity of 3- and 5-amino derivatives of pyridine-2-carboxaldehyde thiosemicarbazone. J. Med. Chem..

[7] Finch R.A., Liu M.-C., Grill S.P., Rose W.C., Loomis R., Vasquez K.M., Cheng Y.-C., Sartorelli A.C. (2000). Triapine (3-aminopyridine-2-carboxaldehyde-thiosemicarbazone): A potent inhibitor of ribonucleotide reductase activity with broad spectrum antitumor activity. Biochem. Pharmacol..

[8] Borges R.H., Paniago E., Beraldo H. (1997). Equilibrium and kinetic studies of iron(II) and iron(III) complexes of some α(*N*)-heterocyclic thiosemicarbazones. Reduction of the iron(III) complexes of 2-formylpyridine thiosemicarbazone and 2-acetylpyridine thiosemicarbazone by cellular thiol-like reducing agents. J. Inorg. Biochem..

[9] Rebolledo A.P., de Lima G.M., Gambi L.N., Speziali N.L., Maia D.F., Pinheiro C.B., Ardisson J.D., Cortés M.E., Beraldo H. (2003). Tin(IV) complexes of 2-benzoylpyridine *N*(4)-phenyl-thiosemicarbazone: Spectral characterization, structural studies and antifungal activity. Appl. Organomet. Chem..

[10] El-Sawaf A.K., West D.X., El-Saied F.A., El-Bahnasawy R.M. (1997). Iron(III), Cobalt(II), Nickel(II), Copper(II) and Zinc(II) complexes of 4-formylantipyrine thiosemicarbazone. Syn. React. Inorg. Met..

[11] Alonso R., Bermejo E., Carballo R., Castiñeiras A., Pérez T. (2002). The supramolecular chemistry of thiosemicarbazones derived from pyrrole: A structural view. J. Mol. Struct..

[12] Brousse B.N., Moglioni A.G., Alho M.M., Álvarez-Larena Á., Moltrasio G.Y., DAccorso N.B. (2002). Behavior of thiosemicarbazones derived from some terpenones under acetylating conditions. Arkivoc.

[13] Kaupp G., Metwally M.A., Amer F.A., Abdel-latif E. (2003). Quantitative Gas-solid diazotization of 3-aminopyrazolo[3,4-*b*]pyridine derivatives and azo dye syntheses by means of solid-solid reactions. Eur. J. Org. Chem..

[14] Abdel-Latif E., Kaupp G., Metwally M.A. (2005). Brand new quantitative solid-state synthesis of *N*-pyrazolylazomethines. J. Chem. Res..

[15] Abdel-Latif E., Metwally M.A. (2007). Waste-Free Solid-state organic syntheses: Solvent-free alkylation, heterocyclization, and azo-coupling reactions. Monatsh. Chem. Chem. Mon..

[16] Bondock S., El-Azap H., Kandeel E.-E.M., Metwally M.A. (2008). Eco-friendly solvent-free synthesis of thiazolylpyrazolederivatives. Monatsh. Chem. Chem. Mon..

[17] Mustafa S.M., Nair V.A., Chittoor J.P., Krishnapillai S. (2004). Synthesis of 1,2,4-triazoles and thiazoles from thiosemicarbazide and its derivatives. Mini Rev. Org. Chem..

[18] Kaupp G. (2003). Solid-state molecular syntheses: complete reactions without auxiliaries based on the new solid-state mechanism. Cryst. Eng. Comm..

[19] Ferguson A.D., Taylor T.P. (1980). Standard methods for the determination of the colour fastness of textiles and leather. J. Soc. Dyers Colour..

[20] Al-Etaibi A.M., El-Apasery M.A., Ibrahim M.R., Al-Awadi N.A. (2012). A facile synthesis of new monoazo disperse dyes derived from 4-hydroxyphenylazopyrazole-5-amines: Evaluation of microwave assisted dyeing behavior. Molecules.

[21] Chipalkatti H.R., Desai N.F., Giles C.H., Macaulay N. (1954). The influence of the substrate upon the light fading of azo dyes. J. Soc. Dyers Colour..

[22] Műller C. (1970). Recent developments in the chemistry of disperse dyes and their intermediate. Am. Dyest. Rep..

[23] Morita M. (1962). Synthesis and antibacterial activity of antipyrine derivatives. IV thiazoles. Yakugaku Zasshi.

